# Supplementary material for: Electrochemical Performance of Orthorhombic CsPbI3 Perovskite in Li-Ion Batteries
Source: Materials (Basel). 2021 Sep 30;14(19):5718. doi: 10.3390/ma14195718 (PMC8510073; doi:10.3390/ma14195718)
Supplement: Supplementary file 1 [file materials-14-05718-s001.zip › materials-1382658-supplementary.pdf]

Supplementary Information

Electrochemical Performance of Orthorhombic $CsPbI_3$ Perovskite in Li-ion batteries

Nahid Kaisar,[a#] Tanmoy Paul,[a#] Po-Wei Chi,[a] Yu-Hsun Su,[a] Anupriya Singh,[b] Chih-Wei Chu,[b] Maw-Kuen Wu,[a] Phillip M. Wu[c]*

[a]Institute of Physics, Academia Sinica, Taipei, Taiwan

[b]Research Center for Applied Science, Academia Sinica, Taipei, Taiwan

[c]Department of Materials and Mineral Resources Engineering, National Taipei University of Technology, Taipei, Taiwan

*Corresponding author
#share equal authorship

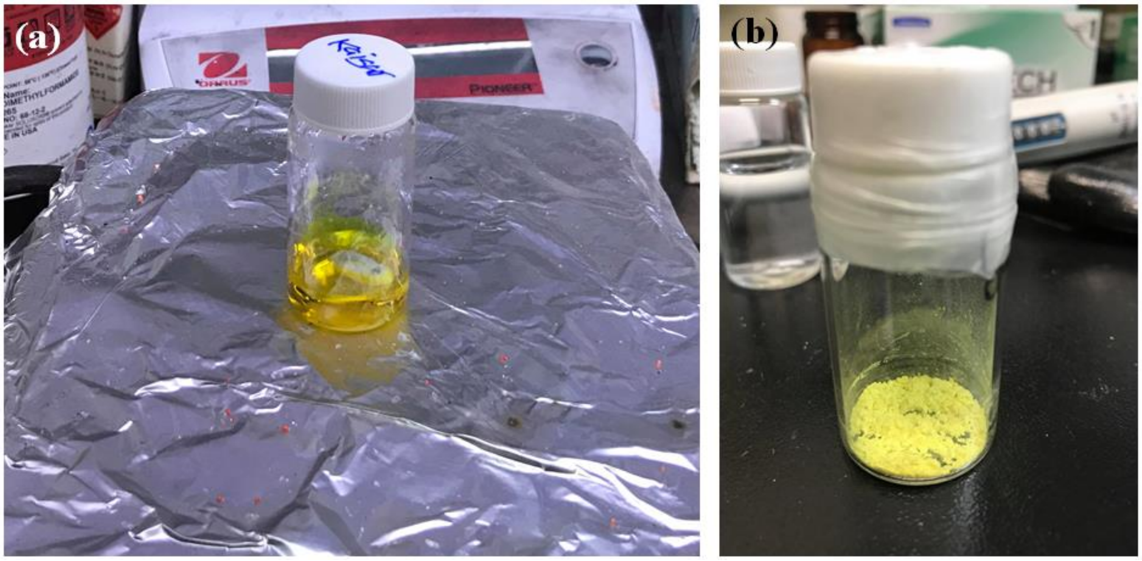

Fig. S1. (a) Photograph of δ-$CsPbI_3$ solution during preparation, and (b) as-prepared δ-$CsPbI_3$ powder.

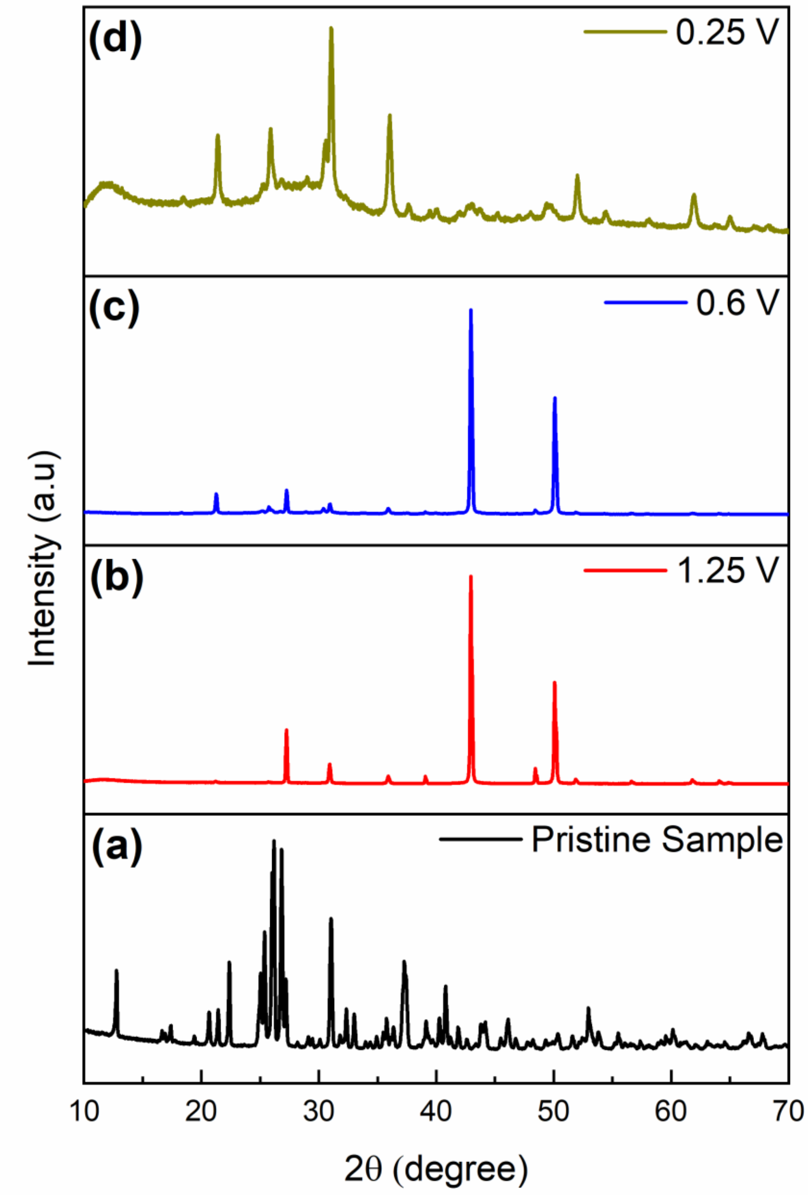

Fig. S2. XRD of fresh $CsPbI_3$ and after one cycle at various voltages during discharging.

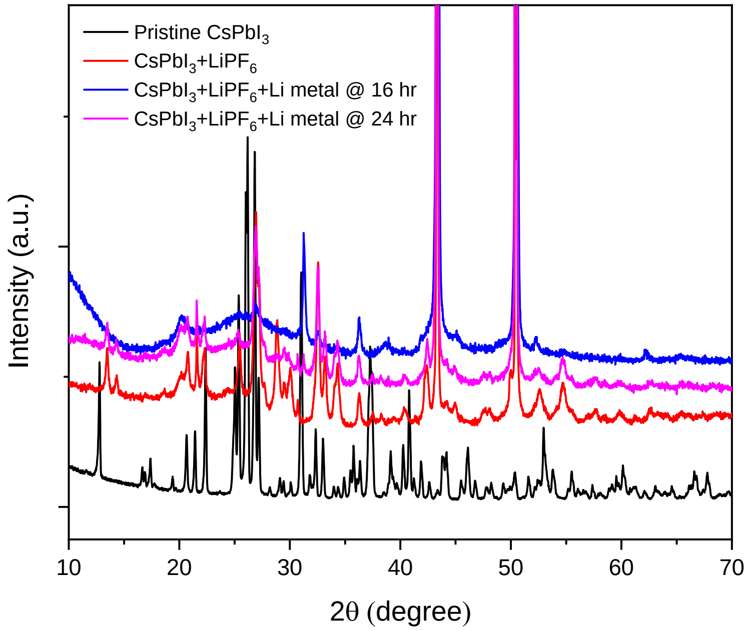

Fig. S3. XRD patterns of pristine $CsPbI_3$, $CsPbI_3$ with $LiPF_6$ salt and $CsPbI_3$ after dipping in electrolyte for different time along with Li metal foil at room temperature.

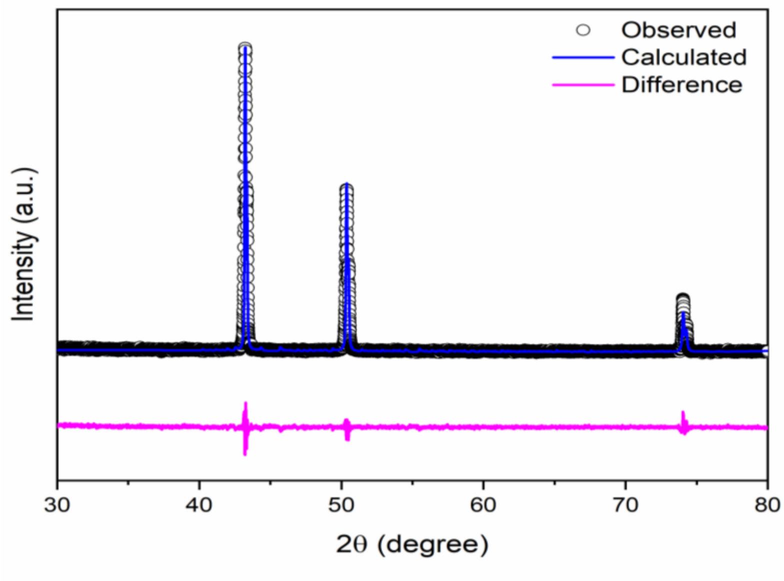

Fig. S4. Rietveld refinement of the XRD pattern of δ-$CsPbI_3$ after 100 cycles at room temperature.

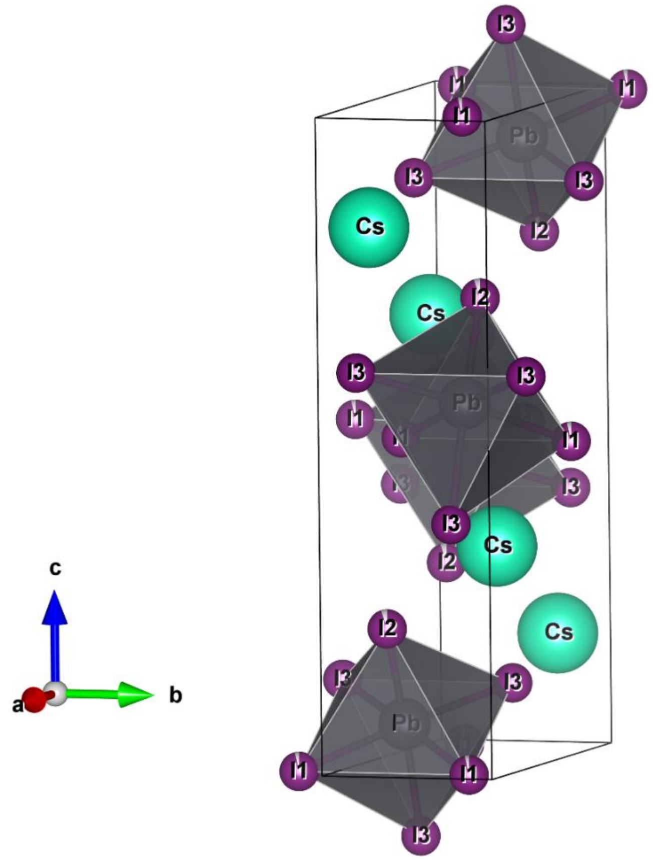

Fig. S5. Orthorhombic structure of δ-$CsPbI_3$. PbI_6 octahedral units are shown in gray color.

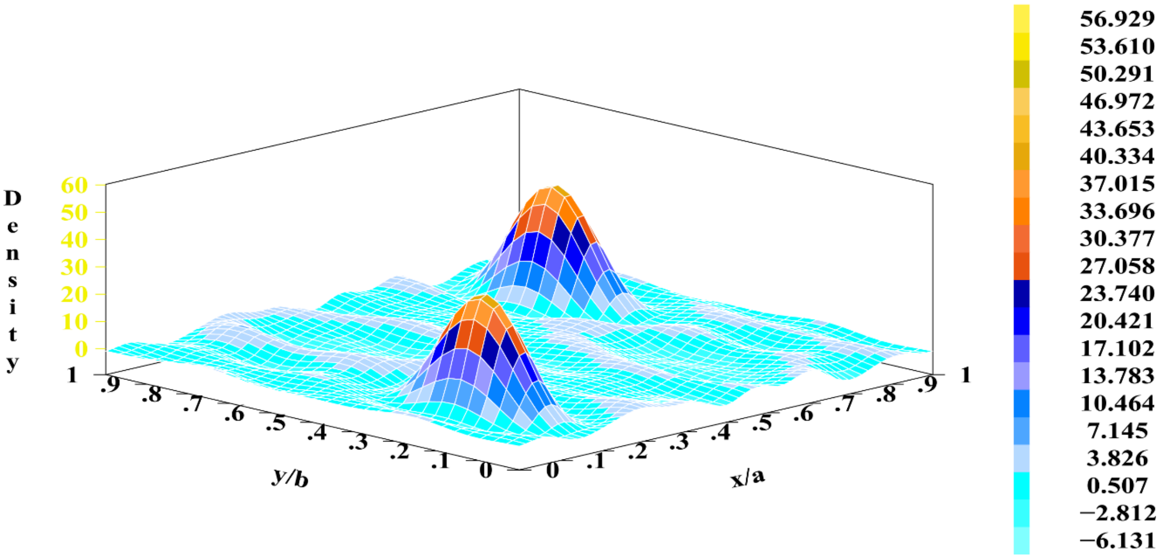

Fig. S6. Electron density distribution of observed structure factors along (110) plane of δ-$CsPbI_3$.

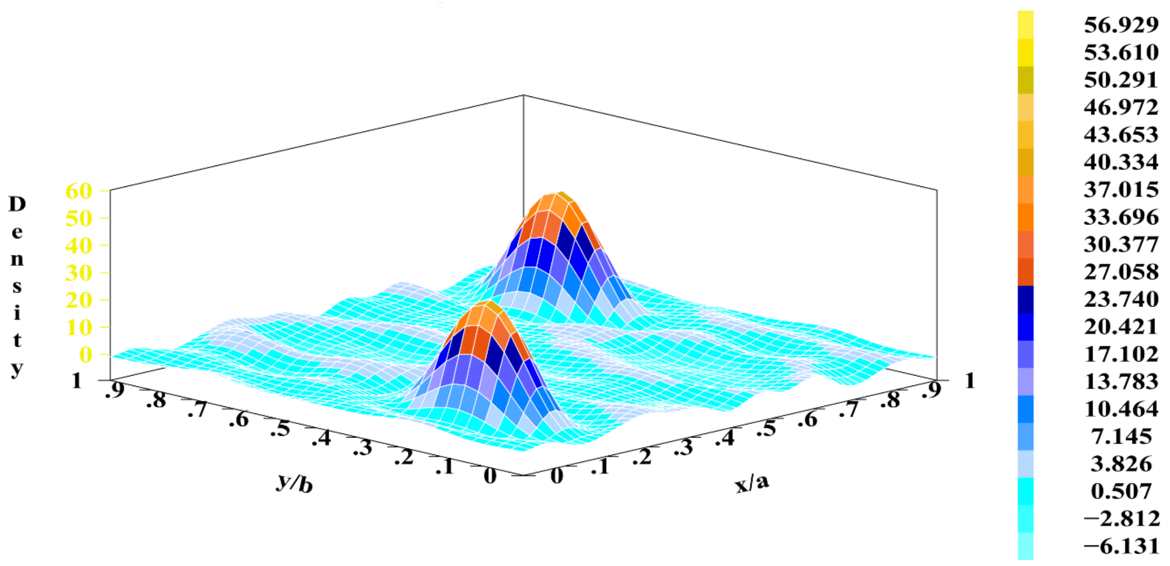

Fig. S7. Electron density distribution of calculated structure factors along (110) plane of δ-$CsPbI_3$.

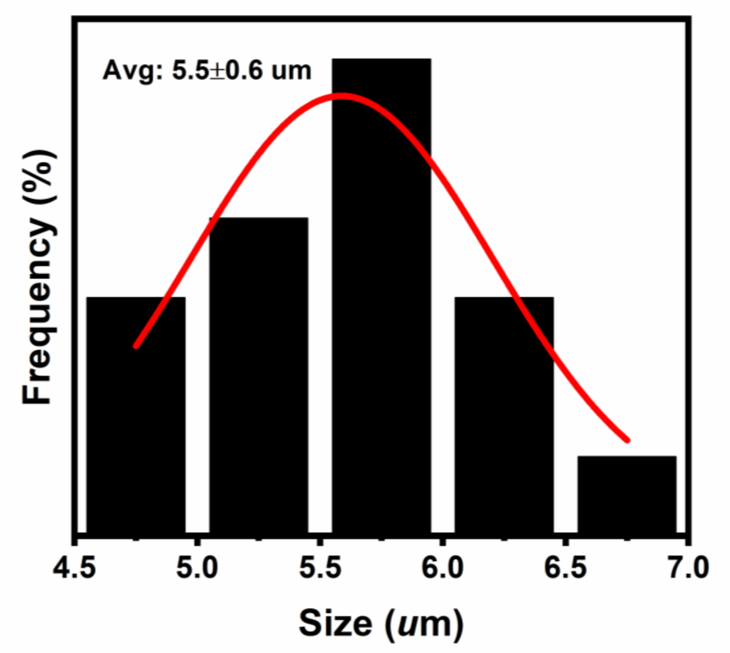

Fig. S8 Particle size distribution for $CsPbI_3$ from SEM image.

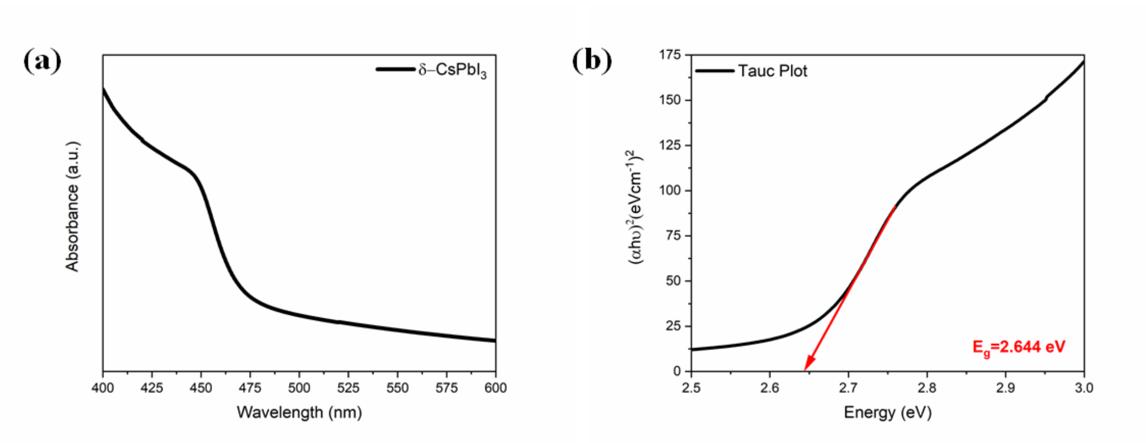

Fig. S9. Optical absorption spectra of $CsPbI_3$ collected by dissolving as-prepared $CsPbI_3$ powder in DMF solvent. (a) UV-Vis spectra and (b) Tauc plot.

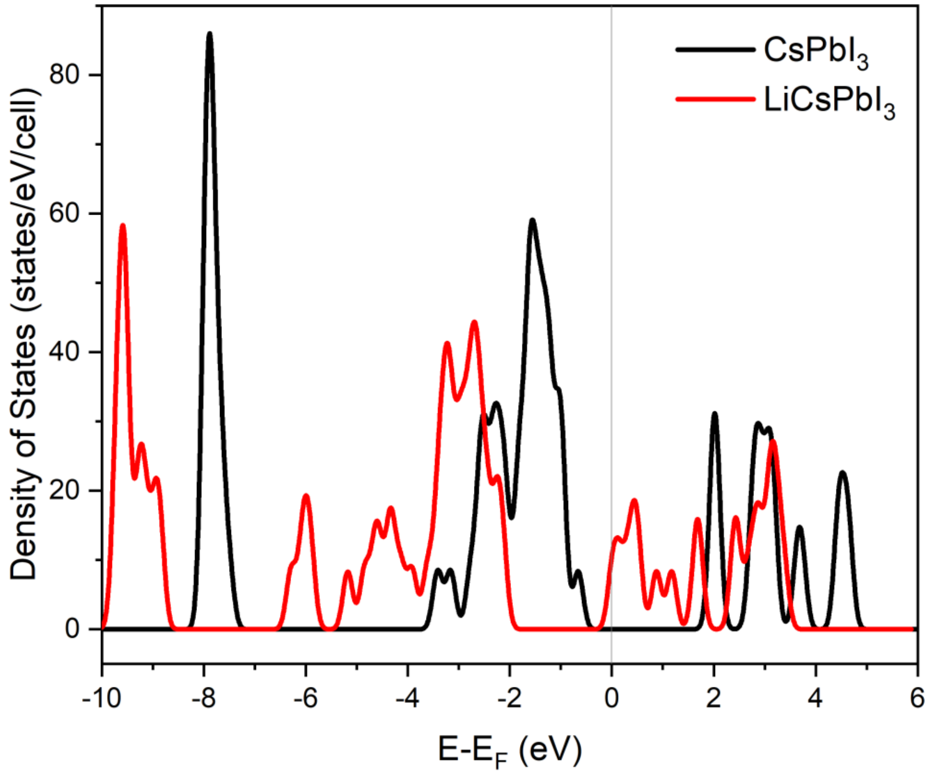

Fig. S10. Total DOS for $CsPbI_3$ and $LiCsPbI_3$. The vertical line represents Fermi level: E_F.

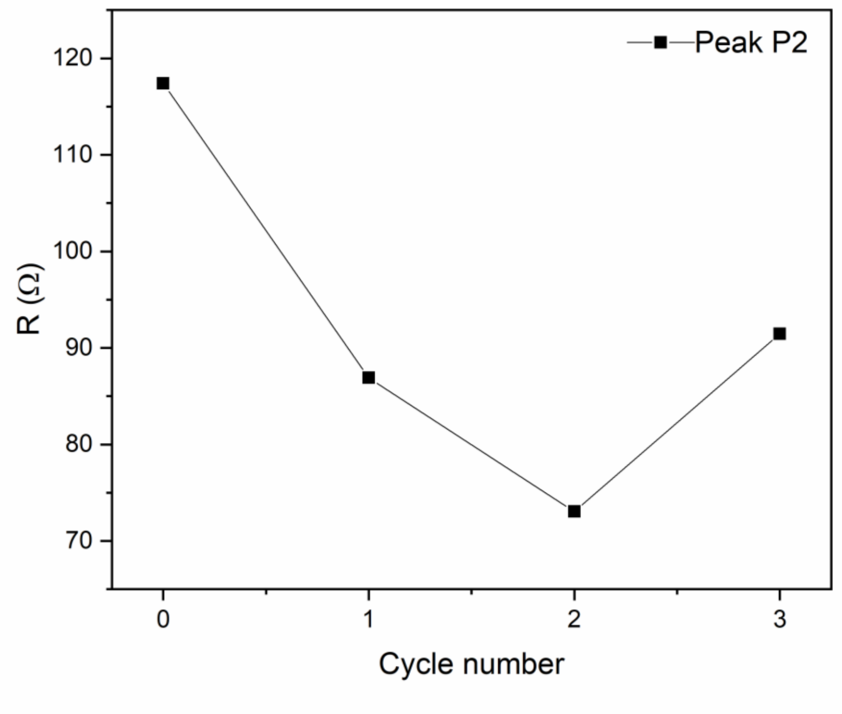

Fig. S11. Variation of the charge transfer resistances along the metal surface with cycles.

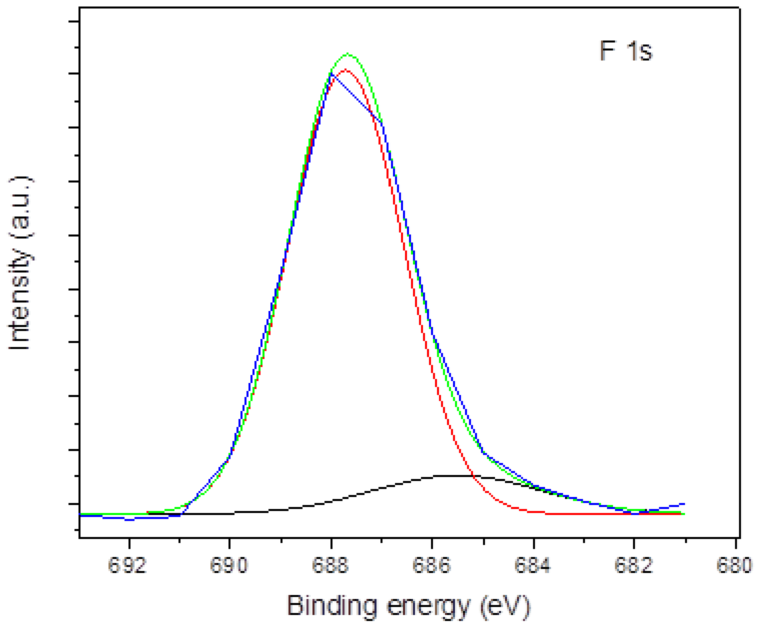

Fig. S12. XPS spectra of F 1s before and after electrochemical discharging process.

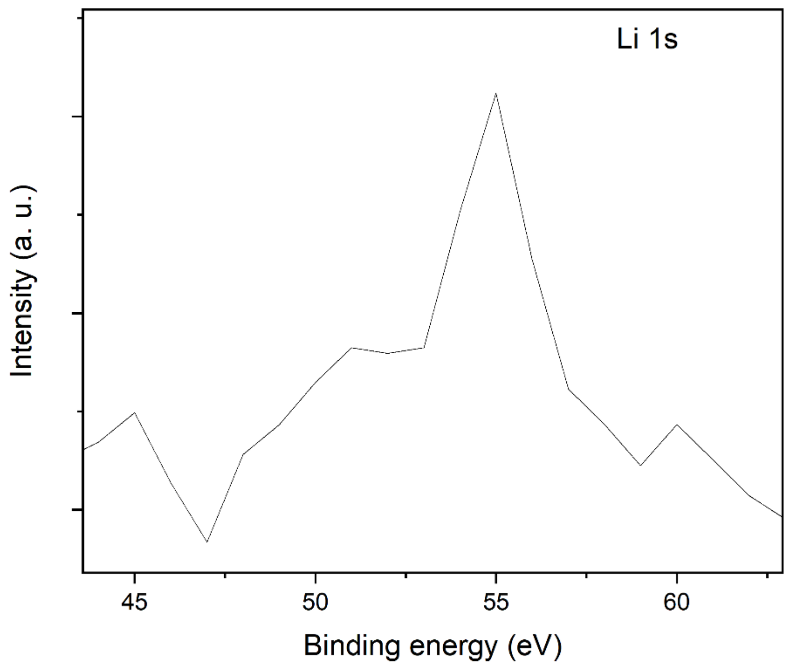

Fig. S13. XPS spectra of Li 1s after electrochemical discharging process.

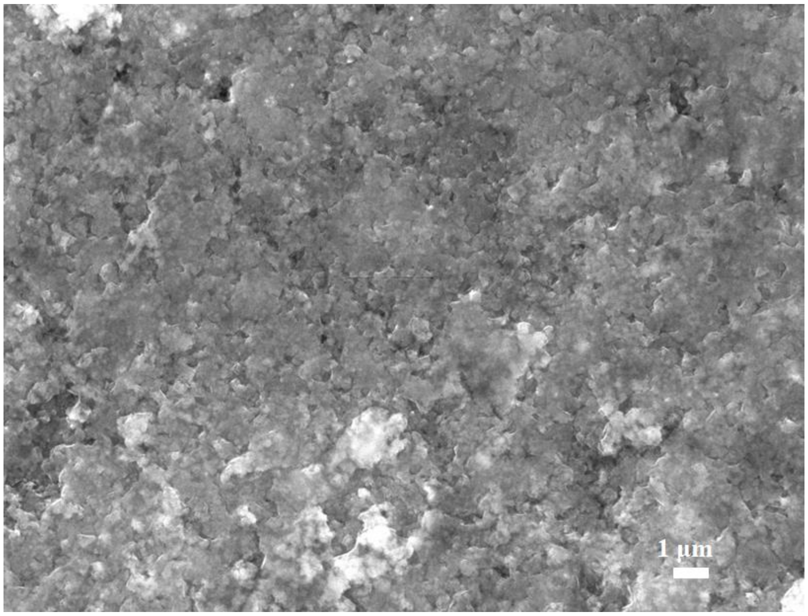

Fig. S14. Top view FESEM image of δ-$CsPbI_3$ electrode after 100 charge-discharge cycles at 40 mA g^{-1}.

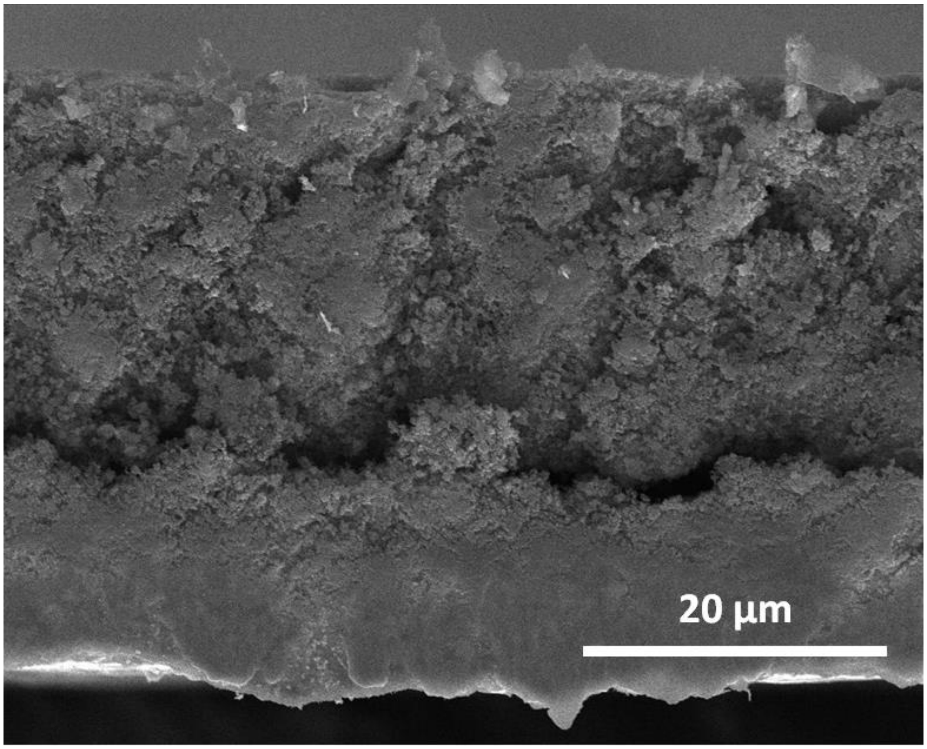

Fig. S15. Cross sectional FESEM image of δ-$CsPbI_3$ electrode after 100 charge-discharge cycles at 40 mA g^{-1}.

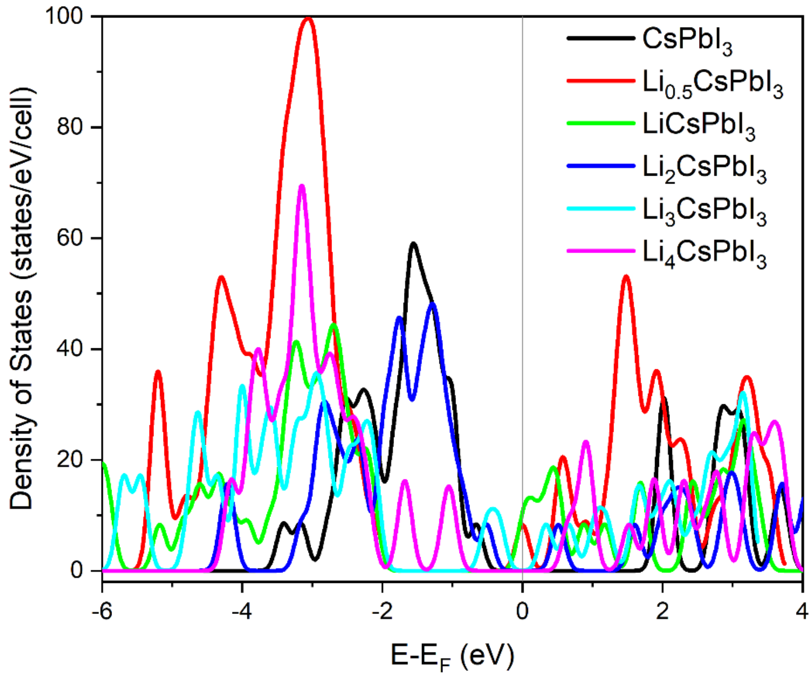

Fig. S16. Total DOS for several lithiated compounds along with pristine $CsPbI_3$. The vertical line represents Fermi level: E_F.

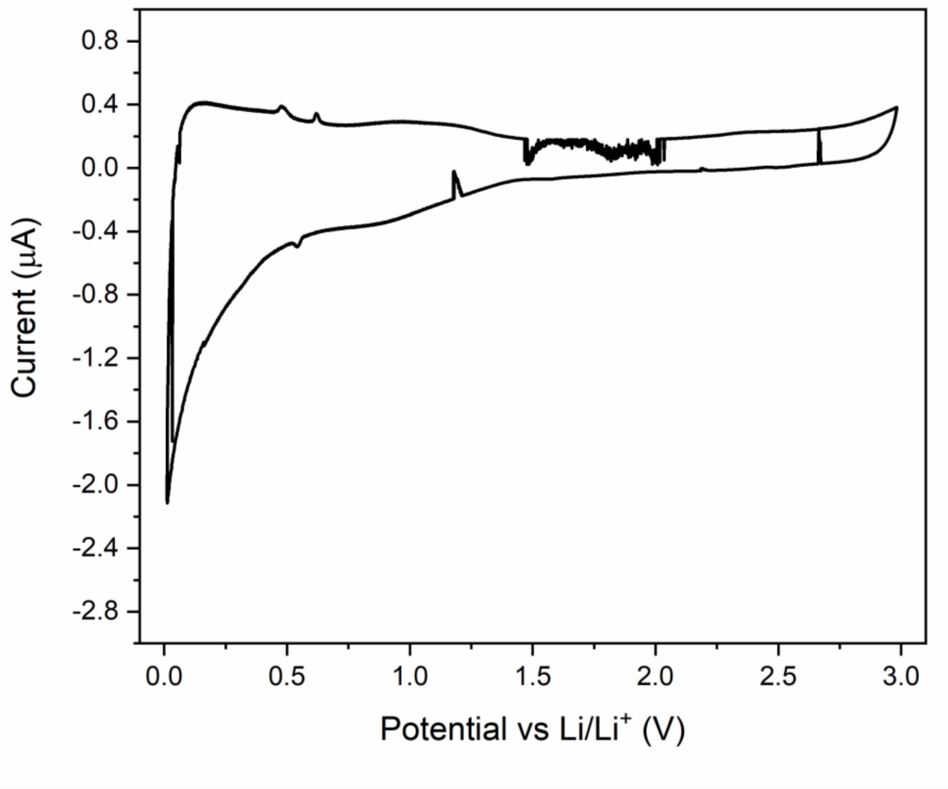

Fig. S17. Cyclic voltammetry trace of a δ-$CsPbI_3$ half-cell after 100 Charge-discharge cycles within a voltage window of 0.1–3.0 V, recorded at a scan rate of 0.01 mV s^{-1}.

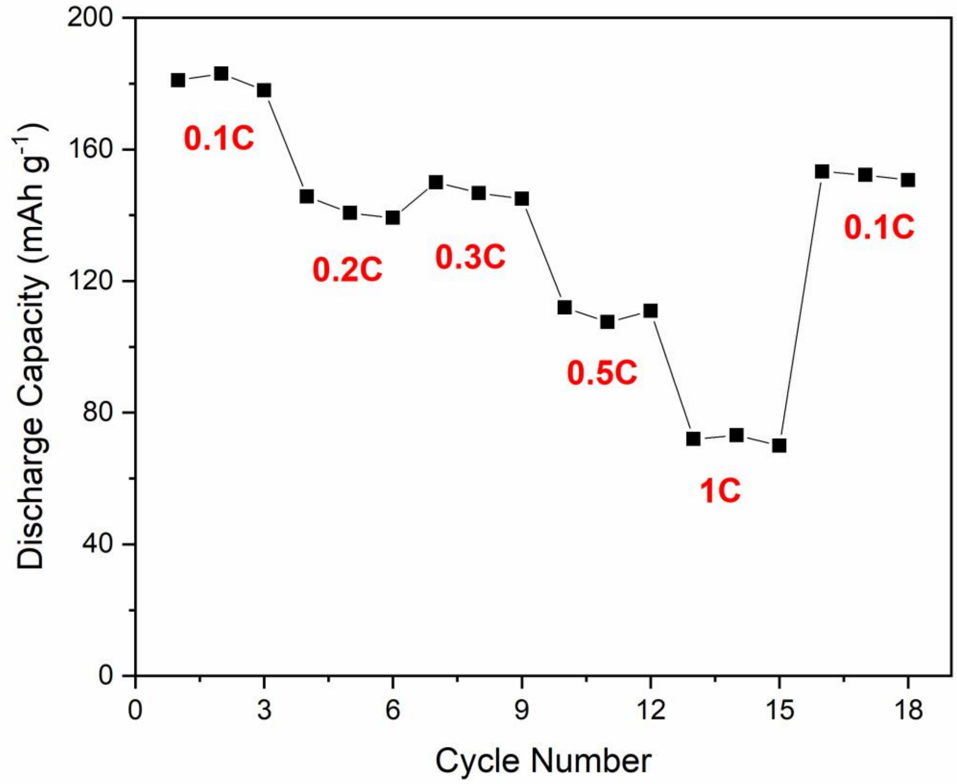

Fig. S18. Cycling performance of $CsPbI_3$ anode materials at different C-rate.

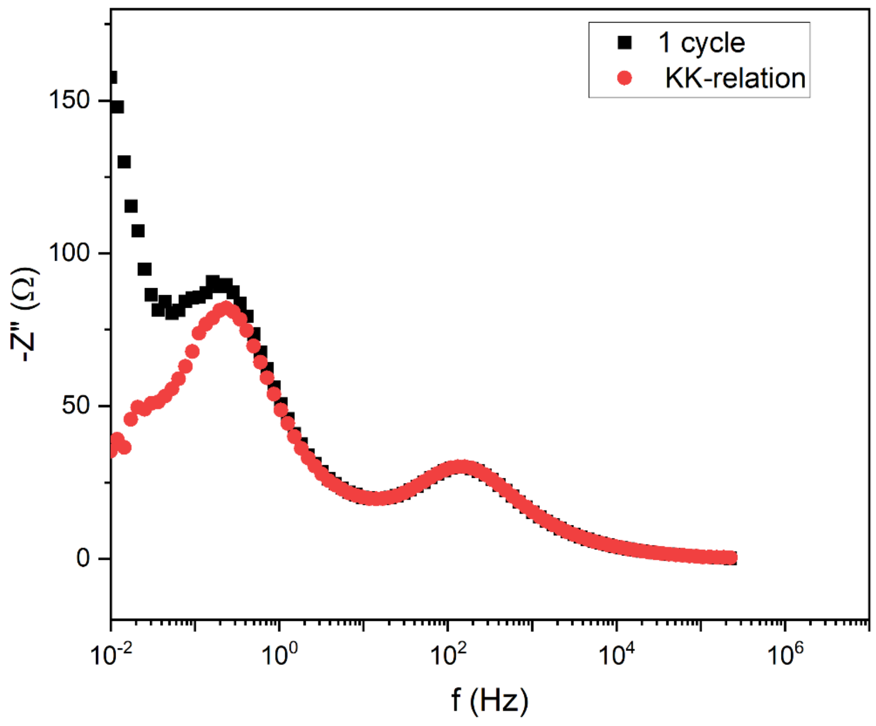

Fig. S19. KK compatibility test.

Table S1. Ground state energy for different configuration of lattice sites with different concentration of lithiation.

Structure	Total Energy (Ry)
4 Li in 4b Wyckoff position_24	-1756.48722
4 Li in 4a Wyckoff position_24	-1756.49541
4 Li in 8d Wyckoff position_24	-1756.53221

8 Li in 8d Wyckoff position_28	-1814.69462
CsPbI3_20	-1697.56303
1 Li in 8d Wyckoff position_21	-1712.83345
